# Cyclo­linopeptide K butanol disolvate monohydrate

**DOI:** 10.1107/S1600536811032363

**Published:** 2011-08-17

**Authors:** Pramodkumar Jadhav, Gabriele Schatte, Shaunivan Labiuk, Peta-Gaye Burnett, Bonnie Li, Denis Okinyo-Owiti, Martin Reaney, Pawel Grochulski, Michel Fodje, Ramaswami Sammynaiken

**Affiliations:** a College of Agriculture & Bioresources, University of Saskatchewan, Saskatoon, Saskatchewan, Canada S7N 5A8; b Saskatchewan Structural Sciences Centre, University of Saskatchewan, Saskatoon, Saskatchewan, Canada S7N 5C9; c Canadian Light Source Inc., University of Saskatchewan, Saskatoon, Saskatchewan, Canada S7N 0X4

## Abstract

The title compound, C_56_H_83_N_9_O_11_S·2C_4_H_10_O·H_2_O, is a butanol–water solvate of the cyclo­linopeptide *cyclo*(Metsulfone^1^-Leu^2^–Ile^3^–Pro^4^–Pro^5^–Phe^6^–Phe^7^–Val^8^–Ile^9^) (henceforth referred to as CLP-K) which was isolated from flax oil. All the amino acid residues are in an l configuration based on the *CORN* rule. The cyclic nona­peptide exhibits eight *trans* peptide bonds and one *cis* peptide bond observed between the two proline residues. The conformation is stabilized by an α- and a β-turn, each containing an N—H⋯O hydrogen bond between the carbonyl group O atom of the first residue and the amide group H atom of the fourth (α-turn) and the third residue (β-turn), repectively. In the crystal, the components of the structure are linked by inter­molecular N—H⋯O and O—H⋯O hydrogen bonds into a two-dimensional network parallel to (001). The –C(H_2_)OH group of one of the butanol solvent mol­ecules is disordered over two sets of sites with refined occupancies of 0.863 (4) and 0.137 (4).

## Related literature

For isolation of cyclo­linopeptides A to B, B to E, F to I and characterization by multi-dimensional NMR spectroscopy, see: Matsumoto *et al.* (2002 ▶); Morita *et al.* (1999 ▶); Matsumoto *et al.* (2001 ▶) respectively. For the isolation of the related cyclo­linopeptide A and its structure determination by single crystal X-ray diffraction in the presence of different solvates, see: Di Blasio *et al.* (1987 ▶, 1989 ▶); Matsumoto *et al.* (2002 ▶); Quail *et al.* (2009 ▶). For the synthesis of cyclo­peptides, see: Rovero *et al.* (1991 ▶); Ghadiri *et al.* (1993 ▶). For immuno-suppressive activity of CLP-A, see: Wieczorek *et al.* (1991 ▶). For the cytoproctective ability of CLP-A, see: Gaymes *et al.* (1997 ▶). For the biomolecular inter­action with human albumin of CLP-A, see: Rempel *et al.* (2010 ▶). For details of the absolute configuration, see: Cahn *et al.* (1966 ▶); Flack & Bernardinelli (2000 ▶); Hooft *et al.* (2008 ▶); *PLATON* (Spek, 2009 ▶).
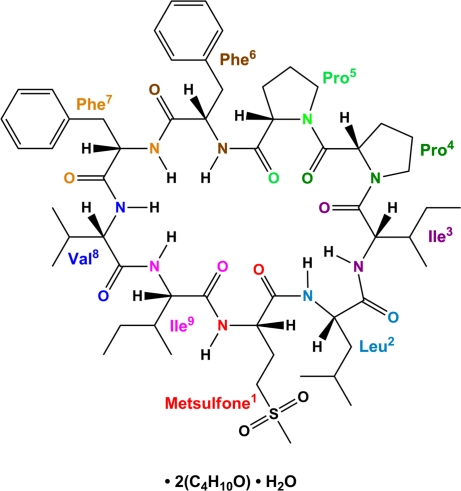


## Experimental

### 

#### Crystal data

C_56_H_83_N_9_O_11_S·2C_4_H_10_O·H_2_O
*M* *_r_* = 1256.63
Orthorhombic, 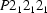

*a* = 11.402 (9) Å
*b* = 23.521 (9) Å
*c* = 25.440 (7) Å
*V* = 6823 (6) Å^3^
*Z* = 4
Synchrotron radiation
λ = 0.68878 Å
μ = 0.12 mm^−1^
*T* = 100 K
0.15 × 0.12 × 0.10 mm

#### Data collection

300mm 16K Rayonix MX300 HE CCD detector with an ACCEL MD2 microdiffractometer
Absorption correction: multi-scan (*SADABS*; Bruker, 2008 ▶) *T*_min_ = 0.983, *T*_max_ = 0.989
549120 measured reflections
15641 independent reflections
14786 reflections with *I* > 2σ(*I*)
*R*_int_ = 0.068

#### Refinement

*R*[*F* ^2^ > 2σ(*F* ^2^)] = 0.039
*wR*(*F* ^2^) = 0.098
*S* = 1.07
15641 reflections
888 parameters
H atoms treated by a mixture of independent and constrained refinement
Δρ_max_ = 0.68 e Å^−3^
Δρ_min_ = −0.27 e Å^−3^
Absolute structure: Flack (1983 ▶), 7070 Friedel pairs
Flack parameter: 0.13 (5)

### 

Data collection: *MXDC* (Canadian Light Source, 2007 ▶); cell refinement: *SAINT* (Bruker, 2008 ▶); data reduction: *SAINT*; program(s) used to solve structure: *SIR2004* (Burla *et al.*, 2005 ▶); program(s) used to refine structure: *SHELXL97* (Sheldrick, 2008 ▶); molecular graphics: *CAMERON* (Watkin *et al.*, 1993 ▶) and *SHELXTL* (Sheldrick, 2008 ▶); software used to prepare material for publication: *publCIF* (Westrip, 2010 ▶).

## Supplementary Material

Crystal structure: contains datablock(s) global, I. DOI: 10.1107/S1600536811032363/lh5292sup1.cif

Structure factors: contains datablock(s) I. DOI: 10.1107/S1600536811032363/lh5292Isup2.hkl

Additional supplementary materials:  crystallographic information; 3D view; checkCIF report

## Figures and Tables

**Table 1:** Hydrogen-bond geometry (Å, °)

*D*—H⋯*A*	*D*—H	H⋯*A*	*D*⋯*A*	*D*—H⋯*A*
N1—H1*D*⋯O3^i^	0.85 (3)	2.21 (3)	3.043 (3)	163 (2)
N2—H2*D*⋯O1^i^	0.81 (2)	2.29 (2)	2.972 (3)	141 (2)
N3—H3*D*⋯O11	0.91 (3)	2.16 (3)	3.005 (2)	154 (2)
N7—H7*D*⋯O5	0.84 (2)	2.14 (2)	2.862 (3)	144 (2)
N8—H8*D*⋯O60	0.82 (2)	2.07 (2)	2.847 (2)	161 (2)
N9—H9*D*⋯O60	0.72 (2)	2.49 (2)	3.203 (2)	169 (2)
O60—H60⋯O5	0.86 (3)	1.91 (3)	2.761 (2)	170 (3)
O70*A*—H70*A*⋯O6^ii^	0.84	2.18	2.795 (2)	130
O80—H80*A*⋯O7^iii^	0.99 (4)	1.95 (4)	2.906 (2)	159 (3)
O80—H80*B*⋯O8^iv^	0.97 (3)	1.95 (3)	2.869 (2)	157 (3)

Symmetry codes: (i) 
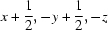
; (ii) 
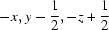
; (iii) 
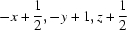
; (iv) 
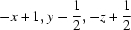
.

**Fig. 1.**
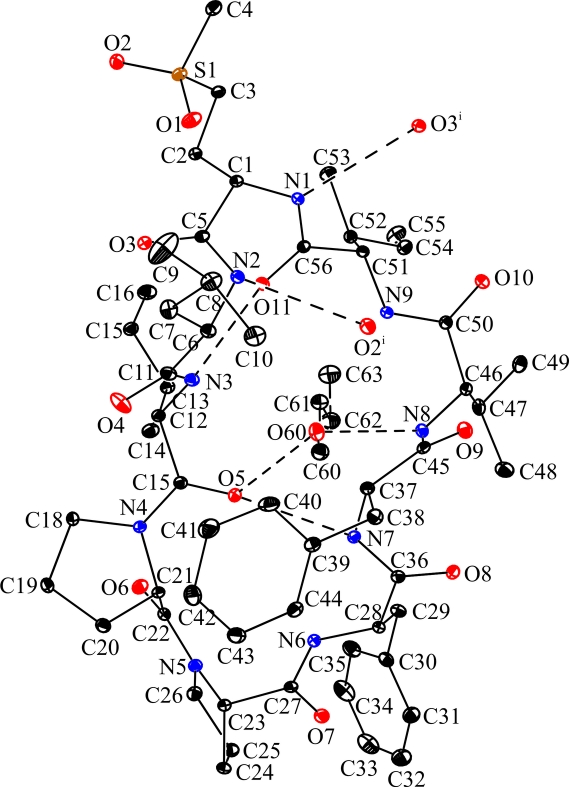
Molecular structure showing the labelling scheme and the inter- and intra-molecular hydrogen bonding. Hydrogen atoms have been omitted for clarity. The non-hydrogen atoms are represented by displacement ellipsoids at the 20% probability level. Symmetry transformations used to generate equivalent atoms: (i) x - 1/2, -y + 1/2, -z.

**Fig. 2.**
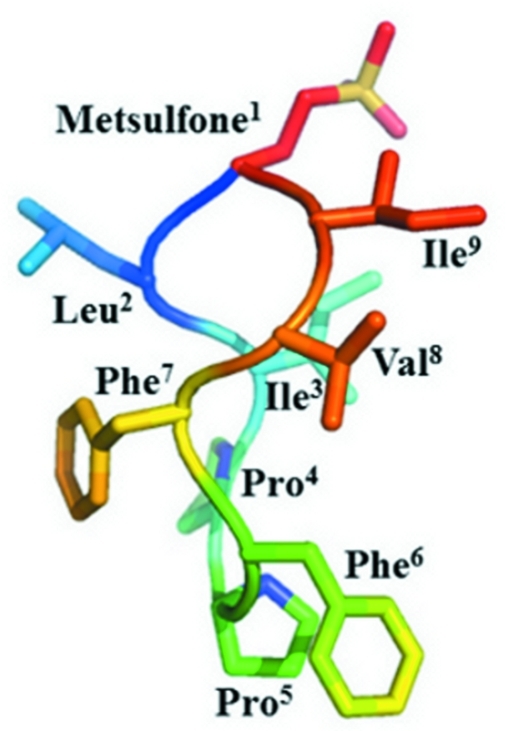
Secondary structure of CLP-K showing the α- and β- turn. The cartoon representation is traced along the backbone of CLP-K. Hydrogen atoms have been omitted for clarity.

## supplementary crystallographic information

### Comment

Flax seeds (bionamial name: *Linum usitatassimum*) contain mostly triglyceride oil (omega-3 fatty acids, and alpha-linolenic acid *etc.*), to lesser amounts lignans and very small amounts of hydrophobic cyclolinopeptides. These *cyclo* nonapeptides have attracted significant interest because of their various biological activities, most importantly because of their immuno-suppressive activity (Wieczorek *et al.*, 1991), their cytoprotective ability, their inhibitory activity toward calcium-dependent activation of T-lymphocyte cell division (Gaymes *et al.*, 1997), and their biomolecular interaction with human albumin (Rempel *et al.*, 2010). The structures of nine different cyclolinopeptides (CLP-A to CLP-I) have been elucidated by two-dimensional FT-NMR spectroscopy (Matsumoto *et al.*, 2002; Morita *et al.*, 1999; Matsumoto *et al.*, 2001). Only structures of CLP-A with different co-crystallized solvent molecules have been determined by single-crystal X-ray diffraction (Di Blasio *et al.*, 1987, 1989); Matsumoto *et al.*, 2002; Quail *et al.* 2009). The crystal structure of *cyclo*(Metsulfone^1^-Leu^2^—Ile^3^—Pro^4^—Pro^5^—Phe^6^—Phe^7^—Val^8^—Ile^9^), CLP-K, was determined as part of our efforts to obtain important information on the biological activity of flax seeds from different locations and strains.

All the amino acid residues in CLP-K are in the *L* configuration based on the *CORN* rule. This is also supported by the *L* configuration of the amino acid residues in the corresponding cyclolinopeptide C, *cyclo*(Metsulfoxide^1^-Leu^2^—Ile^3^—Pro^4^—Pro^5^—Phe^6^—Phe^7^—Val^8^—Ile^9^), determined using derivative chemistry (Morita *et al.*, 1999). Applying the *Cahn-Ingold-Prelog priority rules* (Cahn *et al.*, 1966), the configuration at the chiral α-C atom of each amino acid residue is *S*. The standard uncertainty *u* = 0.05 at Flack parameter *x* = 0.13 implies an enantiopure-sufficient inversion-distinguishing power and together with 2*u* < *x* < 3*u* one cannot say that the crystal is truly enantiopure (Flack & Bernardinelli, 2000). However, the results of the absolute structure determination based on Bayesian statistics on Bijvoet differences (Hooft *et al.*, 2008) using *PLATON* (Spek, 2009) indicate that it is very probable that the absolute configuration has been correctly assigned. The obtained value for the absolute structure parameter *y* was 0.082 (9).

The cyclolinopeptide exhibits eight *trans* peptide bonds with values for ω ranging from 166.49 (14) to 179.30 (14)° (see Table 2) and one *cis* peptide bond observed between the two proline residues (ω = 1.5 (2)°) (see Table 2). The conformation of the cylic peptide is stabilized by an α- and β-turn each containing a hydrogen bond between the carbonyl oxygen of the first residue and the amide hydrogen of the fourth (α-turn) and the third residue (β-turn), repectively. The 5→1 NH···O=C contact bond (α-turn) involves the amide group of Phe^7^ and carbonyl group of Ile^3^ with the two *cis* bonded proline residues Pro^4^ and Pro^5^ being part of this α-turn. The β-turn, a 4→1 NH···O=C contact bond, is formed between the amide group of Ile^3^ and carbonyl group of Ile^9^. The presence of these turns leads to a twisted conformation of CLP-K with an almost V-shaped part at Pro^5^ as depicted in Fig. 2. The side chains of Metsulfone^1^, Leu^2^, Ile^3^, Phe^6^, Phe^7^, Val^8^, Ile^9^ all adopt the *gauche*(+) conformation based on their χ_1_ torsion angles (see Table 2).

One of the oxygen atoms (labelled as O(1)) of the CLP-K methylsufonyl group is linked to a symmetry-related CLP-K unit *via* a S=O···(H)N contact bond (see Table 1). In addition, the CLP-K units are connected *via* the water molecule through hydrogen bonds involving one carbonyl group of each peptide and the hydrogen atoms of the water molecule (see Table 1). These hydrogen bonds together with the S=O···(H)N interconnections are responsible for the formation of a two-dimensional network parallel to (001). The two butanol solvent molecules also form hydrogen bonds with CLP-K (see Table 1). The -C(H_2_)-OH group of one of the butanol solvent molecules is disordered over two sites with refined occupancies of 0.863 (4) and 0.137 (4). The anisotropic displacement parameters of the atoms O71A and O71B, C70A and C70B were constraint to be identical.

### Experimental

Crystals of CLP-K were obtained *via* vapor diffusion, also known as isothermal distillation, at ambient temperature. CLP-K (10 mg) was dissolved in butanol (50 µ*L*) and multiple snowflake-like crystals of CLP-K started to form after fifteen days upon diffusion of *n*-hexane into the sample solution at ambient temperature. The crystalline material was redissolved by adding *n*-butanol (100 uL) and single rod-like crystals of CLP-K, suitable for crystallographic studies, were obtained after eight days.

### Refinement

A suitable single-crystal was removed from the solution, quickly coated with oil (Paratone 8277, Exxon), collected inside a mounted CryoLoop^TM^ (diameter of the nylon fiber: 10 microns; loop diameter 0.1–0.2 mm) and then quickly transferred to the cold stream of the Oxford cryo-jet. The mounted CryoLoop^TM^ had been attached prior to a copper wire (thickness, 0.6 mm; length: 18 mm) attached to a magnetic base using epoxy. Intensity data were collected at 100 K using the beamline 08B1–1 (CMCF-BM; Canadian Light Source, CLS) equipped with a ACCEL MD2 microdiffractometer and a 300 mm 16 K Rayonix MX300 HE CCD detector. The wavelength was set to 0.68878 Å and the distance between the detector and the crystal to 150 mm. The initial screening and data collection was performed with the Macromolecular Crystallography Data Collector (MXDC) graphical user interface. A series of data frames at 1° increments of ω were collected. The integrated intensity data were merged and corrected for absorption using *SADABS* (6, 1 harmonics). The final unit-cell parameters are based upon the refinement of the XYZ weighted centroids of 9248 reflections above 20 σ(I) with 4.67° < 2θ < 54.71°.

The C-bound H atoms, with the exception of the α-C-bound H atoms, were geometrically placed (C–H = 0.98–1.00 Å) and refined as riding with *U_iso_*(H) = 1.2*U_eq_*(parent atom). The hydrogen atoms of the amine groups, the α-C-bound hydrogen atoms and the hydrogen atoms of one of the water molecules were located in the difference Fourier map and were allowed to refine freely. The hydrogen atom of the hydroxyl group in the disordered butanol solvent molecule was geometrically placed (O–H = 0.84 Å) and refined as riding with *U_iso_*(H) = 1.2*U_eq_*(parent atom). The hydrogen atom of the hydroxyl group of the other butanol solvent molecule was located in the difference Fourier map and was allowed to refine freely.

### Crystal data

**Table:**

C_56_H_83_N_9_O_11_S·2C_4_H_10_O·H_2_O	*F*(000) = 2720
*M**_r_* = 1256.63	*D*_x_ = 1.223 Mg m^−^^3^
Orthorhombic, *P*2_1_2_1_2_1_	Synchrotron radiation, λ = 0.68878 Å
Hall symbol: P 2ac 2ab	Cell parameters from 9870 reflections
*a* = 11.402 (9) Å	θ = 2.4–26.5°
*b* = 23.521 (9) Å	µ = 0.12 mm^−^^1^
*c* = 25.440 (7) Å	*T* = 100 K
*V* = 6823 (6) Å^3^	Rod, colourless
*Z* = 4	0.15 × 0.12 × 0.10 mm

### Data collection

**Table:**

300mm 16K Rayonix MX300 HE CCD detector with an ACCEL MD2 microdiffractometer	15641 independent reflections
Radiation source: Beamline 081B-1 at the CLS	14786 reflections with *I* > 2σ(*I*)
double crystal Si(111)	*R*_int_ = 0.068
Detector resolution: 13.8 pixels mm^-1^	θ_max_ = 26.6°, θ_min_ = 1.1°
CCD rotation images, ω scans	*h* = −14→14
Absorption correction: multi-scan (*SADABS*; Bruker, 2008)	*k* = −30→30
*T*_min_ = 0.983, *T*_max_ = 0.989	*l* = −33→33
549120 measured reflections	

### Refinement

**Table:**

Refinement on *F*^2^	Hydrogen site location: inferred from neighbouring sites
Least-squares matrix: full	H atoms treated by a mixture of independent and constrained refinement
*R*[*F*^2^ > 2σ(*F*^2^)] = 0.039	*w* = 1/[σ^2^(*F*_o_^2^) + (0.0428*P*)^2^ + 3.32078*P*] where *P* = (*F*_o_^2^ + 2*F*_c_^2^)/3
*wR*(*F*^2^) = 0.098	(Δ/σ)_max_ = 0.001
*S* = 1.07	Δρ_max_ = 0.68 e Å^−^^3^
15641 reflections	Δρ_min_ = −0.27 e Å^−^^3^
888 parameters	Extinction correction: *SHELXL97* (Sheldrick, 2008), Fc^*^=kFc[1+0.001xFc^2^λ^3^/sin(2θ)]^-1/4^
0 restraints	Extinction coefficient: 0.0066 (3)
Primary atom site location: structure-invariant direct methods	Absolute structure: Flack (1983), 7070 Friedel pairs
Secondary atom site location: difference Fourier map	Flack parameter: 0.13 (5)

### Special details

**Table:**

Geometry. All e.s.d.'s (except the e.s.d. in the dihedral angle between two l.s. planes) are estimated using the full covariance matrix. The cell e.s.d.'s are taken into account individually in the estimation of e.s.d.'s in distances, angles and torsion angles; correlations between e.s.d.'s in cell parameters are only used when they are defined by crystal symmetry. An approximate (isotropic) treatment of cell e.s.d.'s is used for estimating e.s.d.'s involving l.s. planes.
Refinement. Refinement of *F*^2^ against ALL reflections. The weighted *R*-factor *wR* and goodness of fit *S* are based on *F*^2^, conventional *R*-factors *R* are based on *F*, with *F* set to zero for negative *F*^2^. The threshold expression of *F*^2^ > σ(*F*^2^) is used only for calculating *R*-factors(gt) *etc*. and is not relevant to the choice of reflections for refinement. *R*-factors based on *F*^2^ are statistically about twice as large as those based on *F*, and *R*- factors based on ALL data will be even larger.

### Fractional atomic coordinates and isotropic or equivalent isotropic displacement parameters (Å^2^)

**Table:**

	*x*	*y*	*z*	*U*_iso_*/*U*_eq_	Occ. (<1)
S1	−0.06845 (4)	0.127042 (18)	0.090277 (17)	0.02233 (9)	
O1	−0.17707 (11)	0.11209 (5)	0.06398 (5)	0.0271 (3)	
O2	−0.07595 (14)	0.16630 (6)	0.13357 (6)	0.0354 (3)	
O3	−0.08897 (10)	0.29370 (5)	−0.04562 (5)	0.0195 (2)	
O4	−0.13526 (13)	0.43495 (7)	−0.08748 (6)	0.0363 (3)	
O5	−0.00833 (10)	0.52881 (5)	0.04376 (5)	0.0217 (2)	
O6	−0.23894 (11)	0.60979 (5)	0.08609 (5)	0.0254 (3)	
O7	0.14169 (11)	0.73331 (5)	0.00361 (5)	0.0235 (3)	
O8	0.39874 (10)	0.61559 (5)	0.04670 (5)	0.0236 (3)	
O9	0.46130 (11)	0.48642 (5)	−0.00112 (5)	0.0241 (3)	
O10	0.49071 (11)	0.35178 (5)	0.09127 (5)	0.0249 (3)	
O11	0.07474 (10)	0.33509 (5)	0.06117 (5)	0.0205 (2)	
N1	0.17591 (13)	0.26283 (6)	0.02387 (6)	0.0181 (3)	
H1D	0.236 (2)	0.2415 (11)	0.0257 (10)	0.039 (7)*	
N2	0.08840 (13)	0.33307 (6)	−0.05879 (6)	0.0182 (3)	
H2D	0.159 (2)	0.3328 (9)	−0.0533 (9)	0.027 (6)*	
N3	−0.04192 (13)	0.41929 (6)	−0.01064 (6)	0.0191 (3)	
H3D	0.013 (2)	0.3973 (11)	0.0051 (10)	0.040 (7)*	
N4	−0.17491 (12)	0.54896 (6)	0.00023 (6)	0.0187 (3)	
N5	−0.12916 (13)	0.68641 (6)	0.06591 (6)	0.0192 (3)	
N6	0.10867 (13)	0.67134 (6)	0.07003 (6)	0.0198 (3)	
H6D	0.058 (2)	0.6570 (10)	0.0901 (10)	0.035 (6)*	
N7	0.22187 (13)	0.57544 (6)	0.03130 (6)	0.0187 (3)	
H7D	0.149 (2)	0.5777 (9)	0.0358 (8)	0.020 (5)*	
N8	0.35439 (13)	0.48453 (6)	0.07367 (6)	0.0194 (3)	
H8D	0.289 (2)	0.4905 (9)	0.0850 (9)	0.023 (5)*	
N9	0.29706 (13)	0.37135 (6)	0.09964 (6)	0.0184 (3)	
H9D	0.2520 (19)	0.3925 (9)	0.1032 (8)	0.018 (5)*	
C1	0.07734 (15)	0.24387 (7)	−0.00892 (6)	0.0181 (3)	
H1	0.1088 (18)	0.2196 (9)	−0.0359 (8)	0.021 (5)*	
C2	−0.01456 (15)	0.21152 (7)	0.02323 (7)	0.0193 (3)	
H2A	−0.0383	0.2350	0.0537	0.023*	
H2B	−0.0849	0.2051	0.0012	0.023*	
C3	0.03106 (15)	0.15447 (7)	0.04287 (7)	0.0199 (3)	
H3A	0.1095	0.1594	0.0589	0.024*	
H3B	0.0383	0.1276	0.0131	0.024*	
C4	0.00104 (19)	0.06480 (8)	0.11222 (7)	0.0286 (4)	
H4A	0.0806	0.0739	0.1241	0.034*	
H4B	0.0050	0.0373	0.0834	0.034*	
H4C	−0.0437	0.0485	0.1414	0.034*	
C5	0.01818 (14)	0.29271 (7)	−0.03886 (6)	0.0173 (3)	
C6	0.04216 (15)	0.37934 (7)	−0.09113 (7)	0.0202 (3)	
H6	0.1004 (17)	0.4084 (8)	−0.0936 (8)	0.016 (5)*	
C7	0.00408 (17)	0.35989 (8)	−0.14566 (7)	0.0271 (4)	
H7A	−0.0394	0.3913	−0.1628	0.033*	
H7B	−0.0507	0.3275	−0.1417	0.033*	
C8	0.10494 (19)	0.34175 (8)	−0.18180 (7)	0.0302 (4)	
H8	0.1546	0.3132	−0.1630	0.036*	
C9	0.0533 (3)	0.31392 (14)	−0.23131 (10)	0.0657 (9)	
H9A	0.0068	0.3420	−0.2507	0.079*	
H9B	0.0031	0.2820	−0.2211	0.079*	
H9C	0.1172	0.3001	−0.2537	0.079*	
C10	0.18107 (19)	0.39122 (9)	−0.19816 (8)	0.0329 (4)	
H10A	0.1328	0.4197	−0.2161	0.039*	
H10B	0.2425	0.3778	−0.2221	0.039*	
H10C	0.2172	0.4082	−0.1670	0.039*	
C11	−0.05535 (16)	0.41260 (7)	−0.06280 (7)	0.0222 (3)	
C12	−0.13120 (15)	0.44886 (7)	0.02026 (7)	0.0196 (3)	
H12	−0.2079 (16)	0.4453 (8)	0.0012 (7)	0.011 (4)*	
C13	−0.14060 (15)	0.42277 (7)	0.07553 (7)	0.0214 (3)	
H13	−0.0595	0.4167	0.0894	0.026*	
C14	−0.20542 (18)	0.46237 (8)	0.11314 (8)	0.0291 (4)	
H14A	−0.1622	0.4982	0.1163	0.035*	
H14B	−0.2114	0.4444	0.1478	0.035*	
H14C	−0.2843	0.4699	0.0995	0.035*	
C15	−0.20159 (16)	0.36443 (7)	0.07129 (7)	0.0241 (4)	
H15A	−0.2856	0.3705	0.0631	0.029*	
H15B	−0.1665	0.3430	0.0417	0.029*	
C16	−0.1920 (2)	0.32872 (9)	0.12116 (8)	0.0334 (4)	
H16A	−0.1092	0.3240	0.1305	0.040*	
H16B	−0.2273	0.2913	0.1151	0.040*	
H16C	−0.2333	0.3479	0.1499	0.040*	
C17	−0.10061 (14)	0.51252 (7)	0.02211 (6)	0.0185 (3)	
C18	−0.28831 (15)	0.53559 (7)	−0.02517 (8)	0.0242 (4)	
H18A	−0.3384	0.5124	−0.0017	0.029*	
H18B	−0.2763	0.5149	−0.0586	0.029*	
C19	−0.34284 (15)	0.59379 (7)	−0.03497 (8)	0.0255 (4)	
H19A	−0.3925	0.6055	−0.0049	0.031*	
H19B	−0.3911	0.5936	−0.0673	0.031*	
C20	−0.23707 (15)	0.63339 (7)	−0.04102 (7)	0.0232 (3)	
H20A	−0.2586	0.6733	−0.0334	0.028*	
H20B	−0.2038	0.6311	−0.0769	0.028*	
C21	−0.15010 (14)	0.61022 (7)	0.00013 (7)	0.0183 (3)	
H21	−0.0659 (18)	0.6162 (8)	−0.0107 (8)	0.020 (5)*	
C22	−0.17600 (14)	0.63503 (7)	0.05464 (7)	0.0191 (3)	
C23	−0.16410 (17)	0.71832 (8)	0.11377 (7)	0.0248 (4)	
H23A	−0.1416	0.6974	0.1460	0.030*	
H23B	−0.2497	0.7252	0.1143	0.030*	
C24	−0.09648 (17)	0.77413 (7)	0.10923 (7)	0.0261 (4)	
H24A	−0.0194	0.7714	0.1269	0.031*	
H24B	−0.1414	0.8060	0.1248	0.031*	
C25	−0.08167 (16)	0.78212 (7)	0.04989 (7)	0.0231 (3)	
H25A	−0.0147	0.8075	0.0419	0.028*	
H25B	−0.1537	0.7980	0.0339	0.028*	
C26	−0.05830 (15)	0.72114 (7)	0.03017 (7)	0.0200 (3)	
H26	−0.0833 (18)	0.7144 (8)	−0.0064 (8)	0.021 (5)*	
C27	0.07336 (15)	0.70872 (7)	0.03343 (7)	0.0195 (3)	
C28	0.23268 (14)	0.66436 (7)	0.08197 (7)	0.0194 (3)	
H28	0.2752 (17)	0.6983 (8)	0.0714 (7)	0.013 (4)*	
C29	0.25020 (17)	0.65554 (7)	0.14107 (7)	0.0241 (4)	
H29A	0.2097	0.6202	0.1520	0.029*	
H29B	0.3349	0.6508	0.1484	0.029*	
C30	0.20363 (16)	0.70476 (8)	0.17311 (7)	0.0249 (4)	
C31	0.24759 (19)	0.75914 (9)	0.16578 (8)	0.0320 (4)	
H31	0.3056	0.7657	0.1397	0.038*	
C32	0.2071 (2)	0.80423 (9)	0.19648 (10)	0.0423 (5)	
H32	0.2362	0.8416	0.1907	0.051*	
C33	0.1246 (2)	0.79445 (11)	0.23530 (9)	0.0452 (6)	
H33	0.0982	0.8249	0.2567	0.054*	
C34	0.0809 (2)	0.74078 (12)	0.24280 (9)	0.0473 (6)	
H34	0.0248	0.7341	0.2697	0.057*	
C35	0.11831 (19)	0.69604 (9)	0.21119 (8)	0.0353 (5)	
H35	0.0853	0.6593	0.2157	0.042*	
C36	0.29122 (15)	0.61622 (7)	0.05117 (7)	0.0202 (3)	
C37	0.27005 (15)	0.53101 (7)	−0.00266 (7)	0.0196 (3)	
H37	0.2069 (18)	0.5028 (8)	−0.0056 (8)	0.016 (5)*	
C38	0.30238 (16)	0.55437 (8)	−0.05705 (7)	0.0240 (4)	
H38A	0.3555	0.5873	−0.0527	0.029*	
H38B	0.3453	0.5247	−0.0769	0.029*	
C39	0.19690 (15)	0.57258 (7)	−0.08839 (7)	0.0221 (3)	
C40	0.1399 (2)	0.53331 (8)	−0.11996 (8)	0.0338 (4)	
H40	0.1677	0.4953	−0.1212	0.041*	
C41	0.0432 (2)	0.54846 (10)	−0.14979 (10)	0.0432 (5)	
H41	0.0048	0.5208	−0.1709	0.052*	
C42	0.00256 (19)	0.60398 (9)	−0.14873 (8)	0.0356 (4)	
H42	−0.0632	0.6147	−0.1694	0.043*	
C43	0.05820 (18)	0.64371 (8)	−0.11743 (8)	0.0294 (4)	
H43	0.0309	0.6819	−0.1166	0.035*	
C44	0.15384 (16)	0.62784 (7)	−0.08726 (7)	0.0244 (3)	
H44	0.1907	0.6552	−0.0654	0.029*	
C45	0.37290 (15)	0.49933 (7)	0.02332 (7)	0.0192 (3)	
C46	0.43758 (15)	0.44978 (7)	0.10252 (6)	0.0193 (3)	
H46	0.5132 (19)	0.4548 (9)	0.0867 (8)	0.020 (5)*	
C47	0.44064 (16)	0.46801 (8)	0.16082 (7)	0.0245 (4)	
H47	0.3592	0.4651	0.1752	0.029*	
C48	0.4794 (2)	0.53018 (9)	0.16478 (9)	0.0382 (5)	
H48A	0.4822	0.5415	0.2018	0.046*	
H48B	0.4234	0.5543	0.1459	0.046*	
H48C	0.5574	0.5345	0.1491	0.046*	
C49	0.52034 (17)	0.43021 (8)	0.19405 (7)	0.0276 (4)	
H49A	0.6017	0.4347	0.1824	0.033*	
H49B	0.4966	0.3904	0.1900	0.033*	
H49C	0.5139	0.4412	0.2311	0.033*	
C50	0.41129 (14)	0.38636 (7)	0.09661 (6)	0.0188 (3)	
C51	0.26020 (15)	0.31211 (7)	0.10027 (6)	0.0175 (3)	
H51	0.3301 (17)	0.2884 (8)	0.0892 (8)	0.015 (4)*	
C52	0.21596 (15)	0.29313 (7)	0.15511 (6)	0.0202 (3)	
H52	0.1436	0.3154	0.1636	0.024*	
C53	0.18312 (18)	0.23028 (8)	0.15318 (7)	0.0266 (4)	
H53A	0.2479	0.2085	0.1378	0.032*	
H53B	0.1127	0.2254	0.1316	0.032*	
H53C	0.1676	0.2166	0.1889	0.032*	
C54	0.30799 (17)	0.30602 (8)	0.19729 (7)	0.0265 (4)	
H54A	0.3815	0.2860	0.1881	0.032*	
H54B	0.3247	0.3473	0.1969	0.032*	
C55	0.2724 (2)	0.28895 (10)	0.25270 (8)	0.0367 (5)	
H55A	0.1925	0.3022	0.2597	0.044*	
H55B	0.3265	0.3062	0.2781	0.044*	
H55C	0.2754	0.2475	0.2560	0.044*	
C56	0.16265 (14)	0.30451 (7)	0.05998 (6)	0.0168 (3)	
O60	0.12773 (12)	0.47775 (6)	0.12012 (5)	0.0297 (3)	
H60	0.079 (3)	0.4921 (13)	0.0981 (12)	0.057 (8)*	
C61	0.1074 (2)	0.49805 (9)	0.17215 (8)	0.0353 (4)	
H61A	0.0341	0.5206	0.1728	0.042*	
H61B	0.1727	0.5233	0.1828	0.042*	
C62	0.09782 (19)	0.44943 (9)	0.21069 (8)	0.0328 (4)	
H62A	0.1664	0.4241	0.2063	0.039*	
H62B	0.0265	0.4271	0.2026	0.039*	
C63	0.0921 (2)	0.46934 (10)	0.26723 (9)	0.0410 (5)	
H63A	0.1635	0.4917	0.2751	0.049*	
H63B	0.0238	0.4950	0.2713	0.049*	
C64	0.0819 (3)	0.42177 (10)	0.30675 (9)	0.0476 (6)	
H64A	0.0121	0.3990	0.2990	0.057*	
H64B	0.0753	0.4378	0.3422	0.057*	
H64C	0.1518	0.3976	0.3048	0.057*	
O70A	0.1247 (2)	0.09480 (8)	0.31769 (7)	0.0434 (6)	0.863 (4)
H70A	0.1276	0.1154	0.3446	0.052*	0.863 (4)
C71A	0.1982 (3)	0.11838 (12)	0.27765 (10)	0.0463 (8)	0.863 (4)
H71A	0.2799	0.1210	0.2907	0.056*	0.863 (4)
H71B	0.1712	0.1572	0.2688	0.056*	0.863 (4)
O70B	0.2098 (14)	0.0817 (5)	0.3176 (4)	0.0434 (6)	0.137 (4)
H70B	0.2402	0.0965	0.3444	0.052*	0.137 (4)
C71B	0.266 (2)	0.1064 (8)	0.2685 (7)	0.0463 (8)	0.137 (4)
H71C	0.2609	0.1484	0.2679	0.056*	0.137 (4)
H71D	0.3491	0.0947	0.2652	0.056*	0.137 (4)
C72	0.1942 (2)	0.08097 (10)	0.22855 (9)	0.0414 (5)	
H72A	0.1115	0.0774	0.2171	0.050*	
H72B	0.2378	0.1003	0.2001	0.050*	
C73	0.2445 (3)	0.02210 (11)	0.23559 (10)	0.0499 (6)	
H73A	0.2037	0.0032	0.2652	0.060*	
H73B	0.3284	0.0255	0.2451	0.060*	
C74	0.2337 (2)	−0.01505 (10)	0.18712 (11)	0.0489 (6)	
H74A	0.1507	−0.0199	0.1782	0.059*	
H74B	0.2688	−0.0523	0.1942	0.059*	
H74C	0.2747	0.0030	0.1577	0.059*	
O80	0.48874 (14)	0.21728 (6)	0.41673 (6)	0.0350 (3)	
H80A	0.439 (3)	0.2245 (14)	0.4479 (14)	0.075 (10)*	
H80B	0.506 (3)	0.1789 (13)	0.4284 (12)	0.058 (8)*	

### Atomic displacement parameters (Å^2^)

**Table:**

	*U*^11^	*U*^22^	*U*^33^	*U*^12^	*U*^13^	*U*^23^
S1	0.0253 (2)	0.01835 (18)	0.0233 (2)	−0.00384 (16)	0.00250 (17)	−0.00053 (16)
O1	0.0224 (6)	0.0243 (6)	0.0345 (7)	−0.0015 (5)	−0.0004 (5)	0.0024 (5)
O2	0.0452 (8)	0.0292 (7)	0.0320 (7)	−0.0096 (7)	0.0143 (7)	−0.0108 (6)
O3	0.0193 (6)	0.0169 (5)	0.0223 (6)	−0.0013 (4)	−0.0007 (5)	−0.0008 (4)
O4	0.0384 (8)	0.0424 (8)	0.0281 (7)	0.0204 (7)	0.0000 (6)	0.0053 (6)
O5	0.0183 (6)	0.0194 (6)	0.0272 (6)	−0.0022 (5)	−0.0012 (5)	0.0026 (5)
O6	0.0271 (6)	0.0229 (6)	0.0261 (6)	−0.0041 (5)	0.0055 (5)	−0.0012 (5)
O7	0.0219 (6)	0.0206 (6)	0.0280 (6)	0.0004 (5)	0.0029 (5)	0.0033 (5)
O8	0.0185 (6)	0.0193 (6)	0.0329 (7)	−0.0018 (5)	0.0007 (5)	−0.0025 (5)
O9	0.0220 (6)	0.0267 (6)	0.0236 (6)	0.0032 (5)	0.0039 (5)	−0.0030 (5)
O10	0.0203 (6)	0.0220 (6)	0.0323 (7)	0.0033 (5)	0.0014 (5)	−0.0025 (5)
O11	0.0199 (6)	0.0201 (5)	0.0214 (6)	0.0037 (5)	−0.0001 (5)	−0.0029 (5)
N1	0.0155 (6)	0.0160 (6)	0.0230 (7)	0.0016 (5)	−0.0004 (5)	−0.0011 (5)
N2	0.0176 (7)	0.0168 (6)	0.0201 (7)	0.0017 (5)	0.0009 (5)	0.0001 (5)
N3	0.0204 (7)	0.0160 (6)	0.0210 (7)	0.0010 (5)	0.0032 (5)	−0.0001 (5)
N4	0.0174 (6)	0.0141 (6)	0.0246 (7)	0.0001 (5)	−0.0009 (6)	−0.0032 (5)
N5	0.0217 (7)	0.0156 (6)	0.0205 (7)	0.0001 (5)	0.0005 (6)	−0.0023 (5)
N6	0.0171 (7)	0.0178 (7)	0.0246 (7)	−0.0007 (5)	0.0002 (6)	0.0022 (6)
N7	0.0163 (7)	0.0157 (6)	0.0241 (7)	−0.0010 (5)	0.0025 (6)	−0.0003 (5)
N8	0.0158 (7)	0.0194 (7)	0.0229 (7)	0.0013 (5)	0.0019 (6)	0.0002 (5)
N9	0.0170 (7)	0.0160 (6)	0.0222 (7)	0.0025 (6)	0.0005 (5)	0.0006 (5)
C1	0.0190 (8)	0.0151 (7)	0.0202 (8)	0.0004 (6)	−0.0004 (6)	−0.0024 (6)
C2	0.0190 (8)	0.0155 (7)	0.0235 (8)	−0.0002 (6)	0.0003 (6)	−0.0001 (6)
C3	0.0218 (8)	0.0152 (7)	0.0225 (8)	−0.0011 (6)	0.0009 (6)	−0.0003 (6)
C4	0.0374 (10)	0.0231 (9)	0.0253 (9)	−0.0056 (8)	−0.0084 (8)	0.0053 (7)
C5	0.0220 (8)	0.0154 (7)	0.0144 (7)	−0.0003 (6)	0.0015 (6)	−0.0037 (6)
C6	0.0230 (8)	0.0186 (8)	0.0189 (8)	0.0007 (6)	0.0023 (6)	0.0025 (6)
C7	0.0277 (9)	0.0356 (10)	0.0180 (8)	0.0003 (8)	0.0035 (7)	0.0001 (7)
C8	0.0376 (11)	0.0292 (9)	0.0237 (9)	−0.0043 (8)	0.0095 (8)	−0.0055 (7)
C9	0.078 (2)	0.080 (2)	0.0394 (13)	−0.0420 (17)	0.0269 (13)	−0.0311 (14)
C10	0.0383 (11)	0.0341 (10)	0.0262 (9)	−0.0027 (9)	0.0080 (8)	−0.0004 (8)
C11	0.0255 (8)	0.0188 (7)	0.0225 (8)	0.0031 (7)	0.0035 (7)	0.0033 (6)
C12	0.0187 (8)	0.0157 (7)	0.0243 (8)	0.0012 (6)	0.0036 (6)	−0.0026 (6)
C13	0.0221 (8)	0.0182 (8)	0.0239 (8)	−0.0013 (6)	0.0059 (7)	−0.0013 (6)
C14	0.0310 (10)	0.0235 (9)	0.0328 (10)	−0.0010 (8)	0.0115 (8)	−0.0046 (7)
C15	0.0238 (8)	0.0194 (8)	0.0293 (9)	−0.0026 (7)	0.0050 (7)	0.0004 (7)
C16	0.0393 (11)	0.0260 (9)	0.0349 (11)	−0.0017 (8)	0.0117 (9)	0.0059 (8)
C17	0.0189 (8)	0.0170 (7)	0.0198 (8)	0.0007 (6)	0.0042 (6)	−0.0004 (6)
C18	0.0181 (8)	0.0196 (8)	0.0348 (10)	0.0005 (6)	−0.0065 (7)	−0.0035 (7)
C19	0.0193 (8)	0.0229 (9)	0.0343 (10)	0.0039 (7)	−0.0056 (7)	−0.0031 (7)
C20	0.0252 (8)	0.0205 (8)	0.0238 (8)	0.0024 (7)	−0.0030 (7)	−0.0006 (7)
C21	0.0186 (8)	0.0135 (7)	0.0227 (8)	−0.0001 (6)	−0.0004 (6)	−0.0005 (6)
C22	0.0174 (7)	0.0188 (8)	0.0211 (8)	0.0013 (6)	−0.0007 (6)	0.0003 (6)
C23	0.0279 (9)	0.0240 (9)	0.0226 (8)	−0.0001 (7)	0.0013 (7)	−0.0070 (7)
C24	0.0276 (9)	0.0196 (8)	0.0311 (9)	0.0022 (7)	−0.0022 (7)	−0.0063 (7)
C25	0.0240 (8)	0.0143 (7)	0.0311 (9)	0.0039 (6)	−0.0019 (7)	−0.0023 (6)
C26	0.0209 (8)	0.0152 (7)	0.0239 (8)	−0.0006 (6)	−0.0014 (7)	0.0000 (6)
C27	0.0211 (8)	0.0134 (7)	0.0239 (8)	0.0007 (6)	−0.0012 (7)	−0.0020 (6)
C28	0.0180 (8)	0.0156 (7)	0.0247 (8)	0.0012 (6)	−0.0020 (6)	0.0005 (6)
C29	0.0289 (9)	0.0207 (8)	0.0228 (8)	0.0044 (7)	−0.0014 (7)	0.0020 (7)
C30	0.0275 (9)	0.0269 (9)	0.0204 (8)	0.0050 (7)	−0.0033 (7)	0.0005 (7)
C31	0.0357 (10)	0.0286 (10)	0.0318 (10)	−0.0020 (8)	0.0018 (8)	−0.0063 (8)
C32	0.0514 (14)	0.0319 (11)	0.0437 (12)	0.0019 (10)	−0.0018 (10)	−0.0141 (9)
C33	0.0570 (14)	0.0497 (14)	0.0288 (11)	0.0218 (12)	−0.0016 (10)	−0.0130 (10)
C34	0.0513 (14)	0.0628 (16)	0.0277 (10)	0.0218 (13)	0.0092 (10)	0.0041 (10)
C35	0.0373 (11)	0.0379 (11)	0.0308 (10)	0.0084 (9)	0.0054 (8)	0.0092 (9)
C36	0.0215 (8)	0.0169 (8)	0.0221 (8)	0.0003 (6)	−0.0004 (6)	0.0017 (6)
C37	0.0210 (8)	0.0165 (7)	0.0213 (8)	0.0006 (6)	0.0021 (6)	0.0000 (6)
C38	0.0245 (8)	0.0259 (9)	0.0216 (8)	0.0008 (7)	0.0030 (7)	0.0021 (7)
C39	0.0260 (8)	0.0217 (8)	0.0186 (8)	−0.0021 (7)	0.0020 (7)	0.0004 (7)
C40	0.0477 (12)	0.0190 (9)	0.0346 (11)	−0.0013 (8)	−0.0064 (9)	−0.0025 (8)
C41	0.0526 (14)	0.0336 (11)	0.0433 (12)	−0.0055 (10)	−0.0207 (11)	−0.0062 (9)
C42	0.0344 (10)	0.0382 (11)	0.0341 (10)	0.0009 (9)	−0.0120 (9)	0.0008 (9)
C43	0.0324 (10)	0.0243 (9)	0.0313 (10)	0.0013 (8)	0.0028 (8)	0.0017 (7)
C44	0.0252 (8)	0.0209 (8)	0.0272 (9)	−0.0037 (7)	0.0028 (7)	−0.0023 (7)
C45	0.0196 (8)	0.0156 (7)	0.0225 (8)	−0.0034 (6)	−0.0007 (6)	−0.0027 (6)
C46	0.0175 (8)	0.0189 (7)	0.0216 (8)	−0.0007 (6)	−0.0011 (6)	−0.0017 (6)
C47	0.0197 (8)	0.0314 (9)	0.0224 (8)	0.0027 (7)	−0.0008 (7)	−0.0066 (7)
C48	0.0462 (12)	0.0271 (10)	0.0413 (12)	0.0068 (9)	−0.0163 (10)	−0.0155 (9)
C49	0.0267 (9)	0.0344 (10)	0.0218 (8)	−0.0036 (8)	−0.0026 (7)	−0.0005 (7)
C50	0.0203 (8)	0.0200 (8)	0.0162 (7)	−0.0002 (6)	−0.0017 (6)	−0.0018 (6)
C51	0.0191 (8)	0.0154 (7)	0.0180 (8)	0.0009 (6)	−0.0005 (6)	−0.0001 (6)
C52	0.0223 (8)	0.0189 (8)	0.0193 (8)	−0.0001 (7)	−0.0008 (6)	0.0009 (6)
C53	0.0335 (10)	0.0217 (9)	0.0246 (9)	−0.0057 (7)	0.0005 (7)	0.0024 (7)
C54	0.0306 (10)	0.0259 (9)	0.0230 (9)	−0.0034 (7)	−0.0047 (7)	0.0028 (7)
C55	0.0521 (13)	0.0361 (11)	0.0220 (9)	−0.0121 (10)	−0.0057 (9)	0.0018 (8)
C56	0.0182 (7)	0.0153 (7)	0.0169 (7)	−0.0015 (6)	0.0013 (6)	0.0021 (6)
O60	0.0260 (7)	0.0390 (8)	0.0240 (7)	0.0023 (6)	0.0003 (5)	0.0047 (6)
C61	0.0364 (11)	0.0348 (11)	0.0348 (11)	0.0001 (9)	0.0036 (9)	0.0015 (8)
C62	0.0385 (11)	0.0292 (9)	0.0307 (10)	0.0008 (8)	0.0027 (8)	−0.0001 (8)
C63	0.0502 (13)	0.0375 (11)	0.0351 (11)	0.0014 (10)	0.0015 (10)	−0.0061 (9)
C64	0.0701 (17)	0.0427 (13)	0.0301 (11)	−0.0020 (12)	−0.0021 (11)	−0.0019 (9)
O70A	0.0668 (15)	0.0371 (10)	0.0263 (8)	−0.0151 (10)	−0.0020 (9)	0.0046 (7)
C71A	0.077 (2)	0.0348 (14)	0.0267 (12)	−0.0196 (15)	−0.0023 (14)	0.0052 (10)
O70B	0.0668 (15)	0.0371 (10)	0.0263 (8)	−0.0151 (10)	−0.0020 (9)	0.0046 (7)
C71B	0.077 (2)	0.0348 (14)	0.0267 (12)	−0.0196 (15)	−0.0023 (14)	0.0052 (10)
C72	0.0598 (15)	0.0380 (11)	0.0265 (10)	−0.0095 (10)	−0.0087 (10)	0.0084 (9)
C73	0.0518 (15)	0.0502 (14)	0.0476 (14)	0.0056 (12)	−0.0093 (12)	0.0144 (11)
C74	0.0496 (14)	0.0368 (12)	0.0602 (15)	0.0073 (11)	0.0145 (12)	0.0087 (11)
O80	0.0414 (8)	0.0280 (7)	0.0356 (8)	0.0076 (6)	0.0023 (7)	0.0023 (6)

### Geometric parameters (Å, °)

**Table:**

S1—O2	1.4398 (14)	C25—H25B	0.9900
S1—O1	1.4509 (16)	C26—C27	1.532 (3)
S1—C4	1.756 (2)	C26—H26	0.99 (2)
S1—C3	1.7772 (18)	C28—C36	1.530 (2)
O3—C5	1.234 (2)	C28—C29	1.531 (2)
O4—C11	1.225 (2)	C28—H28	0.97 (2)
O5—C17	1.248 (2)	C29—C30	1.512 (3)
O6—C22	1.228 (2)	C29—H29A	0.9900
O7—C27	1.231 (2)	C29—H29B	0.9900
O8—C36	1.231 (2)	C30—C31	1.386 (3)
O9—C45	1.223 (2)	C30—C35	1.388 (3)
O10—C50	1.225 (2)	C31—C32	1.396 (3)
O11—C56	1.234 (2)	C31—H31	0.9500
N1—C56	1.352 (2)	C32—C33	1.384 (4)
N1—C1	1.469 (2)	C32—H32	0.9500
N1—H1D	0.85 (3)	C33—C34	1.370 (4)
N2—C5	1.341 (2)	C33—H33	0.9500
N2—C6	1.463 (2)	C34—C35	1.391 (3)
N2—H2D	0.81 (2)	C34—H34	0.9500
N3—C11	1.345 (2)	C35—H35	0.9500
N3—C12	1.462 (2)	C37—C38	1.534 (2)
N3—H3D	0.91 (3)	C37—C45	1.538 (2)
N4—C17	1.327 (2)	C37—H37	0.98 (2)
N4—C21	1.468 (2)	C38—C39	1.505 (3)
N4—C18	1.479 (2)	C38—H38A	0.9900
N5—C22	1.352 (2)	C38—H38B	0.9900
N5—C26	1.465 (2)	C39—C40	1.386 (3)
N5—C23	1.485 (2)	C39—C44	1.390 (3)
N6—C27	1.343 (2)	C40—C41	1.385 (3)
N6—C28	1.456 (2)	C40—H40	0.9500
N6—H6D	0.84 (3)	C41—C42	1.386 (3)
N7—C36	1.342 (2)	C41—H41	0.9500
N7—C37	1.463 (2)	C42—C43	1.382 (3)
N7—H7D	0.84 (2)	C42—H42	0.9500
N8—C45	1.344 (2)	C43—C44	1.385 (3)
N8—C46	1.452 (2)	C43—H43	0.9500
N8—H8D	0.82 (2)	C44—H44	0.9500
N9—C50	1.352 (2)	C46—C50	1.529 (2)
N9—C51	1.455 (2)	C46—C47	1.544 (2)
N9—H9D	0.72 (2)	C46—H46	0.96 (2)
C1—C2	1.532 (2)	C47—C49	1.527 (3)
C1—C5	1.535 (2)	C47—C48	1.531 (3)
C1—H1	0.96 (2)	C47—H47	1.0000
C2—C3	1.523 (2)	C48—H48A	0.9800
C2—H2A	0.9900	C48—H48B	0.9800
C2—H2B	0.9900	C48—H48C	0.9800
C3—H3A	0.9900	C49—H49A	0.9800
C3—H3B	0.9900	C49—H49B	0.9800
C4—H4A	0.9800	C49—H49C	0.9800
C4—H4B	0.9800	C51—C56	1.523 (2)
C4—H4C	0.9800	C51—C52	1.549 (2)
C6—C7	1.524 (2)	C51—H51	1.01 (2)
C6—C11	1.539 (2)	C52—C53	1.526 (2)
C6—H6	0.95 (2)	C52—C54	1.531 (3)
C7—C8	1.533 (3)	C52—H52	1.0000
C7—H7A	0.9900	C53—H53A	0.9800
C7—H7B	0.9900	C53—H53B	0.9800
C8—C10	1.510 (3)	C53—H53C	0.9800
C8—C9	1.537 (3)	C54—C55	1.521 (3)
C8—H8	1.0000	C54—H54A	0.9900
C9—H9A	0.9800	C54—H54B	0.9900
C9—H9B	0.9800	C55—H55A	0.9800
C9—H9C	0.9800	C55—H55B	0.9800
C10—H10A	0.9800	C55—H55C	0.9800
C10—H10B	0.9800	O60—C61	1.426 (3)
C10—H10C	0.9800	O60—H60	0.86 (3)
C12—C13	1.538 (2)	C61—C62	1.510 (3)
C12—C17	1.538 (2)	C61—H61A	0.9900
C12—H12	1.003 (19)	C61—H61B	0.9900
C13—C14	1.526 (2)	C62—C63	1.514 (3)
C13—C15	1.542 (2)	C62—H62A	0.9900
C13—H13	1.0000	C62—H62B	0.9900
C14—H14A	0.9800	C63—C64	1.509 (3)
C14—H14B	0.9800	C63—H63A	0.9900
C14—H14C	0.9800	C63—H63B	0.9900
C15—C16	1.525 (3)	C64—H64A	0.9800
C15—H15A	0.9900	C64—H64B	0.9800
C15—H15B	0.9900	C64—H64C	0.9800
C16—H16A	0.9800	O70A—C71A	1.431 (3)
C16—H16B	0.9800	O70A—H70A	0.8400
C16—H16C	0.9800	C71A—C72	1.529 (4)
C18—C19	1.524 (2)	C71A—H71A	0.9900
C18—H18A	0.9900	C71A—H71B	0.9900
C18—H18B	0.9900	O70B—C71B	1.52 (2)
C19—C20	1.532 (3)	O70B—H70B	0.8400
C19—H19A	0.9900	C71B—C72	1.436 (19)
C19—H19B	0.9900	C71B—H71C	0.9900
C20—C21	1.542 (2)	C71B—H71D	0.9900
C20—H20A	0.9900	C72—C73	1.509 (3)
C20—H20B	0.9900	C72—H72A	0.9900
C21—C22	1.533 (2)	C72—H72B	0.9900
C21—H21	1.01 (2)	C73—C74	1.516 (4)
C23—C24	1.527 (3)	C73—H73A	0.9900
C23—H23A	0.9900	C73—H73B	0.9900
C23—H23B	0.9900	C74—H74A	0.9800
C24—C25	1.531 (3)	C74—H74B	0.9800
C24—H24A	0.9900	C74—H74C	0.9800
C24—H24B	0.9900	O80—H80A	0.99 (4)
C25—C26	1.543 (2)	O80—H80B	0.97 (3)
C25—H25A	0.9900		
			
O2—S1—O1	117.22 (9)	C36—C28—H28	104.4 (11)
O2—S1—C4	108.57 (10)	C29—C28—H28	108.5 (11)
O1—S1—C4	109.25 (9)	C30—C29—C28	112.32 (14)
O2—S1—C3	108.92 (8)	C30—C29—H29A	109.1
O1—S1—C3	108.67 (9)	C28—C29—H29A	109.1
C4—S1—C3	103.32 (10)	C30—C29—H29B	109.1
C56—N1—C1	121.36 (14)	C28—C29—H29B	109.1
C56—N1—H1D	118.7 (17)	H29A—C29—H29B	107.9
C1—N1—H1D	118.0 (17)	C31—C30—C35	118.92 (18)
C5—N2—C6	121.61 (15)	C31—C30—C29	120.46 (17)
C5—N2—H2D	121.1 (16)	C35—C30—C29	120.60 (18)
C6—N2—H2D	117.3 (16)	C30—C31—C32	120.4 (2)
C11—N3—C12	120.45 (15)	C30—C31—H31	119.8
C11—N3—H3D	116.6 (16)	C32—C31—H31	119.8
C12—N3—H3D	121.1 (16)	C33—C32—C31	119.9 (2)
C17—N4—C21	120.75 (14)	C33—C32—H32	120.1
C17—N4—C18	127.14 (14)	C31—C32—H32	120.1
C21—N4—C18	112.10 (13)	C34—C33—C32	120.0 (2)
C22—N5—C26	125.77 (14)	C34—C33—H33	120.0
C22—N5—C23	121.32 (15)	C32—C33—H33	120.0
C26—N5—C23	112.02 (13)	C33—C34—C35	120.3 (2)
C27—N6—C28	120.66 (15)	C33—C34—H34	119.8
C27—N6—H6D	118.5 (17)	C35—C34—H34	119.8
C28—N6—H6D	119.7 (17)	C30—C35—C34	120.4 (2)
C36—N7—C37	120.78 (15)	C30—C35—H35	119.8
C36—N7—H7D	119.1 (14)	C34—C35—H35	119.8
C37—N7—H7D	119.8 (14)	O8—C36—N7	122.91 (16)
C45—N8—C46	121.69 (15)	O8—C36—C28	119.37 (15)
C45—N8—H8D	115.9 (16)	N7—C36—C28	117.70 (15)
C46—N8—H8D	121.2 (16)	N7—C37—C38	111.54 (14)
C50—N9—C51	121.94 (15)	N7—C37—C45	112.25 (14)
C50—N9—H9D	120.9 (17)	C38—C37—C45	112.20 (14)
C51—N9—H9D	117.0 (17)	N7—C37—H37	104.6 (11)
N1—C1—C2	111.78 (14)	C38—C37—H37	110.5 (11)
N1—C1—C5	113.02 (13)	C45—C37—H37	105.3 (11)
C2—C1—C5	109.65 (14)	C39—C38—C37	112.82 (15)
N1—C1—H1	107.4 (12)	C39—C38—H38A	109.0
C2—C1—H1	110.0 (12)	C37—C38—H38A	109.0
C5—C1—H1	104.7 (12)	C39—C38—H38B	109.0
C3—C2—C1	112.28 (14)	C37—C38—H38B	109.0
C3—C2—H2A	109.1	H38A—C38—H38B	107.8
C1—C2—H2A	109.1	C40—C39—C44	118.00 (17)
C3—C2—H2B	109.1	C40—C39—C38	119.48 (17)
C1—C2—H2B	109.1	C44—C39—C38	122.51 (16)
H2A—C2—H2B	107.9	C41—C40—C39	121.30 (19)
C2—C3—S1	108.93 (12)	C41—C40—H40	119.3
C2—C3—H3A	109.9	C39—C40—H40	119.3
S1—C3—H3A	109.9	C40—C41—C42	119.86 (19)
C2—C3—H3B	109.9	C40—C41—H41	120.1
S1—C3—H3B	109.9	C42—C41—H41	120.1
H3A—C3—H3B	108.3	C43—C42—C41	119.66 (19)
S1—C4—H4A	109.5	C43—C42—H42	120.2
S1—C4—H4B	109.5	C41—C42—H42	120.2
H4A—C4—H4B	109.5	C42—C43—C44	119.90 (18)
S1—C4—H4C	109.5	C42—C43—H43	120.1
H4A—C4—H4C	109.5	C44—C43—H43	120.1
H4B—C4—H4C	109.5	C43—C44—C39	121.27 (17)
O3—C5—N2	121.66 (16)	C43—C44—H44	119.4
O3—C5—C1	121.24 (15)	C39—C44—H44	119.4
N2—C5—C1	117.06 (15)	O9—C45—N8	123.35 (16)
N2—C6—C7	113.03 (14)	O9—C45—C37	122.02 (16)
N2—C6—C11	112.05 (14)	N8—C45—C37	114.53 (15)
C7—C6—C11	111.91 (15)	N8—C46—C50	111.81 (14)
N2—C6—H6	108.6 (12)	N8—C46—C47	110.13 (14)
C7—C6—H6	110.6 (12)	C50—C46—C47	111.71 (14)
C11—C6—H6	99.8 (12)	N8—C46—H46	107.8 (12)
C6—C7—C8	114.57 (16)	C50—C46—H46	104.8 (12)
C6—C7—H7A	108.6	C47—C46—H46	110.4 (13)
C8—C7—H7A	108.6	C49—C47—C48	110.37 (16)
C6—C7—H7B	108.6	C49—C47—C46	112.55 (15)
C8—C7—H7B	108.6	C48—C47—C46	109.58 (16)
H7A—C7—H7B	107.6	C49—C47—H47	108.1
C10—C8—C7	112.46 (17)	C48—C47—H47	108.1
C10—C8—C9	108.81 (18)	C46—C47—H47	108.1
C7—C8—C9	108.83 (19)	C47—C48—H48A	109.5
C10—C8—H8	108.9	C47—C48—H48B	109.5
C7—C8—H8	108.9	H48A—C48—H48B	109.5
C9—C8—H8	108.9	C47—C48—H48C	109.5
C8—C9—H9A	109.5	H48A—C48—H48C	109.5
C8—C9—H9B	109.5	H48B—C48—H48C	109.5
H9A—C9—H9B	109.5	C47—C49—H49A	109.5
C8—C9—H9C	109.5	C47—C49—H49B	109.5
H9A—C9—H9C	109.5	H49A—C49—H49B	109.5
H9B—C9—H9C	109.5	C47—C49—H49C	109.5
C8—C10—H10A	109.5	H49A—C49—H49C	109.5
C8—C10—H10B	109.5	H49B—C49—H49C	109.5
H10A—C10—H10B	109.5	O10—C50—N9	123.05 (16)
C8—C10—H10C	109.5	O10—C50—C46	120.92 (15)
H10A—C10—H10C	109.5	N9—C50—C46	115.99 (15)
H10B—C10—H10C	109.5	N9—C51—C56	108.42 (13)
O4—C11—N3	122.69 (17)	N9—C51—C52	112.31 (13)
O4—C11—C6	121.04 (16)	C56—C51—C52	109.55 (14)
N3—C11—C6	116.06 (15)	N9—C51—H51	107.2 (11)
N3—C12—C13	110.50 (14)	C56—C51—H51	108.9 (11)
N3—C12—C17	108.76 (13)	C52—C51—H51	110.4 (11)
C13—C12—C17	112.12 (14)	C53—C52—C54	112.50 (15)
N3—C12—H12	107.9 (11)	C53—C52—C51	109.27 (14)
C13—C12—H12	110.3 (11)	C54—C52—C51	110.55 (14)
C17—C12—H12	107.1 (10)	C53—C52—H52	108.1
C14—C13—C12	111.31 (15)	C54—C52—H52	108.1
C14—C13—C15	111.63 (15)	C51—C52—H52	108.1
C12—C13—C15	108.82 (14)	C52—C53—H53A	109.5
C14—C13—H13	108.3	C52—C53—H53B	109.5
C12—C13—H13	108.3	H53A—C53—H53B	109.5
C15—C13—H13	108.3	C52—C53—H53C	109.5
C13—C14—H14A	109.5	H53A—C53—H53C	109.5
C13—C14—H14B	109.5	H53B—C53—H53C	109.5
H14A—C14—H14B	109.5	C55—C54—C52	114.49 (16)
C13—C14—H14C	109.5	C55—C54—H54A	108.6
H14A—C14—H14C	109.5	C52—C54—H54A	108.6
H14B—C14—H14C	109.5	C55—C54—H54B	108.6
C16—C15—C13	113.53 (16)	C52—C54—H54B	108.6
C16—C15—H15A	108.9	H54A—C54—H54B	107.6
C13—C15—H15A	108.9	C54—C55—H55A	109.5
C16—C15—H15B	108.9	C54—C55—H55B	109.5
C13—C15—H15B	108.9	H55A—C55—H55B	109.5
H15A—C15—H15B	107.7	C54—C55—H55C	109.5
C15—C16—H16A	109.5	H55A—C55—H55C	109.5
C15—C16—H16B	109.5	H55B—C55—H55C	109.5
H16A—C16—H16B	109.5	O11—C56—N1	122.02 (15)
C15—C16—H16C	109.5	O11—C56—C51	120.54 (15)
H16A—C16—H16C	109.5	N1—C56—C51	117.44 (14)
H16B—C16—H16C	109.5	C61—O60—H60	111 (2)
O5—C17—N4	121.67 (15)	O60—C61—C62	111.15 (17)
O5—C17—C12	120.22 (15)	O60—C61—H61A	109.4
N4—C17—C12	118.10 (15)	C62—C61—H61A	109.4
N4—C18—C19	103.72 (14)	O60—C61—H61B	109.4
N4—C18—H18A	111.0	C62—C61—H61B	109.4
C19—C18—H18A	111.0	H61A—C61—H61B	108.0
N4—C18—H18B	111.0	C61—C62—C63	112.68 (18)
C19—C18—H18B	111.0	C61—C62—H62A	109.1
H18A—C18—H18B	109.0	C63—C62—H62A	109.1
C18—C19—C20	103.97 (14)	C61—C62—H62B	109.1
C18—C19—H19A	111.0	C63—C62—H62B	109.1
C20—C19—H19A	111.0	H62A—C62—H62B	107.8
C18—C19—H19B	111.0	C64—C63—C62	114.02 (19)
C20—C19—H19B	111.0	C64—C63—H63A	108.7
H19A—C19—H19B	109.0	C62—C63—H63A	108.7
C19—C20—C21	102.90 (14)	C64—C63—H63B	108.7
C19—C20—H20A	111.2	C62—C63—H63B	108.7
C21—C20—H20A	111.2	H63A—C63—H63B	107.6
C19—C20—H20B	111.2	C63—C64—H64A	109.5
C21—C20—H20B	111.2	C63—C64—H64B	109.5
H20A—C20—H20B	109.1	H64A—C64—H64B	109.5
N4—C21—C22	109.56 (13)	C63—C64—H64C	109.5
N4—C21—C20	102.95 (13)	H64A—C64—H64C	109.5
C22—C21—C20	110.84 (13)	H64B—C64—H64C	109.5
N4—C21—H21	108.6 (11)	C71A—O70A—H70A	109.5
C22—C21—H21	112.2 (11)	O70A—C71A—C72	109.9 (2)
C20—C21—H21	112.2 (11)	O70A—C71A—H71A	109.7
O6—C22—N5	121.66 (16)	C72—C71A—H71A	109.7
O6—C22—C21	121.19 (15)	O70A—C71A—H71B	109.7
N5—C22—C21	117.13 (14)	C72—C71A—H71B	109.7
N5—C23—C24	103.72 (14)	H71A—C71A—H71B	108.2
N5—C23—H23A	111.0	C71B—O70B—H70B	109.5
C24—C23—H23A	111.0	C72—C71B—O70B	100.5 (13)
N5—C23—H23B	111.0	C72—C71B—H71C	111.7
C24—C23—H23B	111.0	O70B—C71B—H71C	111.7
H23A—C23—H23B	109.0	C72—C71B—H71D	111.7
C23—C24—C25	103.64 (14)	O70B—C71B—H71D	111.7
C23—C24—H24A	111.0	H71C—C71B—H71D	109.4
C25—C24—H24A	111.0	C71B—C72—C73	94.6 (10)
C23—C24—H24B	111.0	C73—C72—C71A	114.8 (2)
C25—C24—H24B	111.0	C71B—C72—H72A	141.9
H24A—C24—H24B	109.0	C73—C72—H72A	108.6
C24—C25—C26	103.04 (14)	C71A—C72—H72A	108.6
C24—C25—H25A	111.2	C71B—C72—H72B	92.3
C26—C25—H25A	111.2	C73—C72—H72B	108.6
C24—C25—H25B	111.2	C71A—C72—H72B	108.6
C26—C25—H25B	111.2	H72A—C72—H72B	107.5
H25A—C25—H25B	109.1	C72—C73—C74	113.7 (2)
N5—C26—C27	113.61 (14)	C72—C73—H73A	108.8
N5—C26—C25	102.78 (14)	C74—C73—H73A	108.8
C27—C26—C25	109.21 (14)	C72—C73—H73B	108.8
N5—C26—H26	109.7 (12)	C74—C73—H73B	108.8
C27—C26—H26	107.6 (12)	H73A—C73—H73B	107.7
C25—C26—H26	114.0 (12)	C73—C74—H74A	109.5
O7—C27—N6	123.03 (16)	C73—C74—H74B	109.5
O7—C27—C26	119.81 (15)	H74A—C74—H74B	109.5
N6—C27—C26	117.15 (15)	C73—C74—H74C	109.5
N6—C28—C36	113.61 (14)	H74A—C74—H74C	109.5
N6—C28—C29	110.30 (14)	H74B—C74—H74C	109.5
C36—C28—C29	110.23 (14)	H80A—O80—H80B	92 (2)
N6—C28—H28	109.5 (11)		
			
C56—N1—C1—C2	−69.93 (18)	C25—C26—C27—N6	109.82 (17)
C56—N1—C1—C5	54.3 (2)	C27—N6—C28—C36	−94.07 (18)
N1—C1—C2—C3	−68.07 (17)	C27—N6—C28—C29	141.59 (16)
C5—C1—C2—C3	165.78 (13)	N6—C28—C29—C30	−59.00 (19)
C1—C2—C3—S1	168.11 (11)	C36—C28—C29—C30	174.75 (15)
O2—S1—C3—C2	−60.58 (14)	C28—C29—C30—C31	−59.7 (2)
O1—S1—C3—C2	68.19 (13)	C28—C29—C30—C35	121.90 (19)
C4—S1—C3—C2	−175.86 (12)	C35—C30—C31—C32	0.4 (3)
C6—N2—C5—O3	−2.1 (2)	C29—C30—C31—C32	−178.02 (19)
C6—N2—C5—C1	175.65 (14)	C30—C31—C32—C33	1.5 (3)
N1—C1—C5—O3	−142.96 (15)	C31—C32—C33—C34	−1.4 (4)
C2—C1—C5—O3	−17.5 (2)	C32—C33—C34—C35	−0.6 (4)
N1—C1—C5—N2	39.23 (19)	C31—C30—C35—C34	−2.4 (3)
C2—C1—C5—N2	164.67 (14)	C29—C30—C35—C34	176.02 (19)
C5—N2—C6—C7	−71.9 (2)	C33—C34—C35—C30	2.6 (4)
C5—N2—C6—C11	55.7 (2)	C37—N7—C36—O8	−7.5 (3)
N2—C6—C7—C8	−68.8 (2)	C37—N7—C36—C28	174.28 (14)
C11—C6—C7—C8	163.58 (15)	N6—C28—C36—O8	160.55 (16)
C6—C7—C8—C10	−68.9 (2)	C29—C28—C36—O8	−75.1 (2)
C6—C7—C8—C9	170.50 (19)	N6—C28—C36—N7	−21.2 (2)
C12—N3—C11—O4	7.0 (3)	C29—C28—C36—N7	103.17 (18)
C12—N3—C11—C6	−178.29 (14)	C36—N7—C37—C38	−72.1 (2)
N2—C6—C11—O4	−149.19 (17)	C36—N7—C37—C45	54.8 (2)
C7—C6—C11—O4	−21.0 (2)	N7—C37—C38—C39	−67.15 (19)
N2—C6—C11—N3	36.0 (2)	C45—C37—C38—C39	165.93 (14)
C7—C6—C11—N3	164.14 (15)	C37—C38—C39—C40	−88.6 (2)
C11—N3—C12—C13	146.05 (15)	C37—C38—C39—C44	91.7 (2)
C11—N3—C12—C17	−90.48 (18)	C44—C39—C40—C41	0.3 (3)
N3—C12—C13—C14	163.62 (14)	C38—C39—C40—C41	−179.4 (2)
C17—C12—C13—C14	42.1 (2)	C39—C40—C41—C42	0.6 (4)
N3—C12—C13—C15	−72.95 (18)	C40—C41—C42—C43	−0.7 (4)
C17—C12—C13—C15	165.55 (14)	C41—C42—C43—C44	−0.2 (3)
C14—C13—C15—C16	−69.6 (2)	C42—C43—C44—C39	1.1 (3)
C12—C13—C15—C16	167.21 (15)	C40—C39—C44—C43	−1.2 (3)
C21—N4—C17—O5	0.8 (2)	C38—C39—C44—C43	178.52 (17)
C18—N4—C17—O5	−178.07 (16)	C46—N8—C45—O9	−2.3 (2)
C21—N4—C17—C12	−179.28 (14)	C46—N8—C45—C37	174.08 (14)
C18—N4—C17—C12	1.8 (3)	N7—C37—C45—O9	−138.38 (16)
N3—C12—C17—O5	−63.5 (2)	C38—C37—C45—O9	−11.8 (2)
C13—C12—C17—O5	59.0 (2)	N7—C37—C45—N8	45.15 (19)
N3—C12—C17—N4	116.64 (17)	C38—C37—C45—N8	171.69 (14)
C13—C12—C17—N4	−120.86 (17)	C45—N8—C46—C50	−89.61 (19)
C17—N4—C18—C19	171.41 (16)	C45—N8—C46—C47	145.58 (15)
C21—N4—C18—C19	−7.56 (19)	N8—C46—C47—C49	175.97 (15)
N4—C18—C19—C20	28.20 (18)	C50—C46—C47—C49	51.1 (2)
C18—C19—C20—C21	−38.05 (18)	N8—C46—C47—C48	−60.82 (19)
C17—N4—C21—C22	−77.03 (19)	C50—C46—C47—C48	174.31 (15)
C18—N4—C21—C22	102.01 (16)	C51—N9—C50—O10	4.2 (3)
C17—N4—C21—C20	164.98 (15)	C51—N9—C50—C46	−173.40 (14)
C18—N4—C21—C20	−15.97 (18)	N8—C46—C50—O10	140.00 (16)
C19—C20—C21—N4	32.78 (16)	C47—C46—C50—O10	−96.07 (19)
C19—C20—C21—C22	−84.29 (16)	N8—C46—C50—N9	−42.4 (2)
C26—N5—C22—O6	−176.63 (16)	C47—C46—C50—N9	81.56 (18)
C23—N5—C22—O6	−8.3 (2)	C50—N9—C51—C56	−131.06 (16)
C26—N5—C22—C21	1.7 (2)	C50—N9—C51—C52	107.76 (17)
C23—N5—C22—C21	170.00 (15)	N9—C51—C52—C53	−178.01 (14)
N4—C21—C22—O6	−19.1 (2)	C56—C51—C52—C53	61.46 (18)
C20—C21—C22—O6	93.81 (19)	N9—C51—C52—C54	−53.68 (19)
N4—C21—C22—N5	162.59 (14)	C56—C51—C52—C54	−174.22 (14)
C20—C21—C22—N5	−84.48 (18)	C53—C52—C54—C55	−57.6 (2)
C22—N5—C23—C24	−176.92 (15)	C51—C52—C54—C55	179.91 (16)
C26—N5—C23—C24	−7.11 (19)	C1—N1—C56—O11	−12.7 (2)
N5—C23—C24—C25	28.18 (18)	C1—N1—C56—C51	166.44 (14)
C23—C24—C25—C26	−38.51 (18)	N9—C51—C56—O11	−53.7 (2)
C22—N5—C26—C27	−89.54 (19)	C52—C51—C56—O11	69.17 (19)
C23—N5—C26—C27	101.19 (17)	N9—C51—C56—N1	127.18 (15)
C22—N5—C26—C25	152.60 (16)	C52—C51—C56—N1	−109.94 (16)
C23—N5—C26—C25	−16.67 (18)	O60—C61—C62—C63	172.53 (18)
C24—C25—C26—N5	33.60 (17)	C61—C62—C63—C64	179.8 (2)
C24—C25—C26—C27	−87.33 (17)	O70B—C71B—C72—C73	−72.0 (13)
C28—N6—C27—O7	9.6 (2)	O70B—C71B—C72—C71A	58.0 (13)
C28—N6—C27—C26	−169.37 (14)	O70A—C71A—C72—C71B	−122.2 (14)
N5—C26—C27—O7	176.69 (14)	O70A—C71A—C72—C73	−65.0 (4)
C25—C26—C27—O7	−69.2 (2)	C71B—C72—C73—C74	−155.3 (9)
N5—C26—C27—N6	−4.3 (2)	C71A—C72—C73—C74	177.1 (2)

### Hydrogen-bond geometry (Å, °)

**Table:**

*D*—H···*A*	*D*—H	H···*A*	*D*···*A*	*D*—H···*A*
N1—H1D···O3^i^	0.85 (3)	2.21 (3)	3.043 (3)	163 (2)
N2—H2D···O1^i^	0.81 (2)	2.29 (2)	2.972 (3)	141 (2)
N3—H3D···O11	0.91 (3)	2.16 (3)	3.005 (2)	154 (2)
N7—H7D···O5	0.84 (2)	2.14 (2)	2.862 (3)	144 (2)
N8—H8D···O60	0.82 (2)	2.07 (2)	2.847 (2)	161 (2)
N9—H9D···O60	0.72 (2)	2.49 (2)	3.203 (2)	169 (2)
O60—H60···O5	0.86 (3)	1.91 (3)	2.761 (2)	170 (3)
O70A—H70A···O6^ii^	0.84	2.18	2.795 (2)	130
O80—H80A···O7^iii^	0.99 (4)	1.95 (4)	2.906 (2)	159 (3)
O80—H80B···O8^iv^	0.97 (3)	1.95 (3)	2.869 (2)	157 (3)

Symmetry codes: (i) *x*+1/2, −*y*+1/2, −*z*; (ii) −*x*, *y*−1/2, −*z*+1/2; (iii) −*x*+1/2, −*y*+1, *z*+1/2; (iv) −*x*+1, *y*−1/2, −*z*+1/2.

### Table 2 Backbone torsion angles φ, ψ, ω and side chain torsion angle χ1 (°) in CLP-K

**Table:**

	Met sulfone^1^	Leu^2^	Ile^3^	Pro^4^	Pro^5^	Phe^6^	Phe^7^	Val^8^	Ile^9^
									
φ	54.3 (2)	55.7 (2)	-90.48 (18)	-77.03 (19)	-89.54 (19)	-94.07 (18)	54.8 (2)	-89.61 (18)	-131.06 (16)
ψ	39.23 (19)	36.0 (2)	116.64 (17)	162.59 (14)	-4.3 (2)	-21.2 (2)	45.15 (19)	-42.4 (2)	127.18 (15)
ω	166.44 (14)	175.65 (14)	-178.29 (14)	-179.28 (14)	1.6 (2)	-169.37 (14)	174.28 (14)	174.08 (14)	-173.40 (14)
χ1	-68.07 (17)	-68.8 (2)	-72.95 (18)	32.78 (16)	33.60 (17)	-59.00 (19)	-67.15 (19)	-60.82 (19)	-53.68 (19)
